# Prediction by genetic MATS of 4CMenB vaccine strain coverage of invasive meningococcal serogroup B isolates circulating in Taiwan between 2003 and 2020

**DOI:** 10.1128/msphere.00220-24

**Published:** 2024-05-16

**Authors:** Alessandro Muzzi, Min-Chi Lu, Elena Mori, Alessia Biolchi, Tiffany Fu, Laura Serino

**Affiliations:** 1 GSK, Siena, Italy; 2 School of Medicine, China Medical University, Taichung, Taiwan; 3 GSK, Taipei, Taiwan; The University of Texas Medical Branch at Galveston, Galveston, Texas, USA

**Keywords:** 4CMenB, genetic Meningococcal Antigen Typing System, meningococcal disease, serogroup B, strain coverage, Taiwan

## Abstract

*Neisseria meningitidis* serogroup B (NmB) strains have diverse antigens, necessitating methods for predicting meningococcal serogroup B (MenB) vaccine strain coverage. The genetic Meningococcal Antigen Typing System (gMATS), a correlate of MATS estimates, predicts strain coverage by the 4-component MenB (4CMenB) vaccine in cultivable and non-cultivable NmB isolates. In Taiwan, 134 invasive, disease-causing NmB isolates were collected in 2003–2020 (23.1%, 4.5%, 5.2%, 29.8%, and 37.3% from individuals aged ≤11 months, 12–23 months, 2–4 years, 5–29 years, and ≥30 years, respectively). NmB isolates were characterized by whole-genome sequencing and vaccine antigen genotyping, and 4CMenB strain coverage was predicted using gMATS. Analysis of phylogenetic relationships with 502 global NmB genomes showed that most isolates belonged to three global hyperinvasive clonal complexes: ST-4821 (27.6%), ST-32 (23.9%), and ST-41/44 (14.9%). Predicted strain coverage by gMATS was 62.7%, with 27.6% isolates covered, 2.2% not covered, and 66.4% unpredictable by gMATS. Age group coverage point estimates ranged from 42.9% (2–4 years) to 66.1% (≤11 months). Antigen coverage estimates and percentages predicted as covered/not covered were highly variable, with higher estimates for isolates with one or more gMATS-positive antigens than for isolates positive for one 4CMenB antigen. In conclusion, this first study on NmB strain coverage by 4CMenB in Taiwan shows 62.7% coverage by gMATS, with predictable coverage for 29.8% of isolates. These could be underestimated since the gMATS calculation does not consider synergistic mechanisms associated with simultaneous antibody binding to multiple targets elicited by multicomponent vaccines or the contributions of minor outer membrane vesicle vaccine components.

**IMPORTANCE:**

Meningococcal diseases, caused by the bacterium *Neisseria meningitidis* (meningococcus), include meningitis and septicemia. Although rare, invasive meningococcal disease is often severe and can be fatal. Nearly all cases are caused by six meningococcal serogroups (types), including meningococcal serogroup B. Vaccines are available against meningococcal serogroup B, but the antigens targeted by these vaccines have highly variable genetic features and expression levels, so the effectiveness of vaccination may vary depending on the strains circulating in particular countries. It is therefore important to test meningococcal serogroup B strains isolated from specific populations to estimate the percentage of bacterial strains that a vaccine can protect against (vaccine strain coverage). Meningococcal isolates were collected in Taiwan between 2003 and 2020, of which 134 were identified as serogroup B. We did further investigations on these isolates, including using a method (called gMATS) to predict vaccine strain coverage by the 4-component meningococcal serogroup B vaccine (4CMenB).

## INTRODUCTION

The *Neisseria meningitidis* (Nm) bacterium causes invasive meningococcal disease (IMD) by crossing the epithelium of the nasopharynx and passing into the bloodstream (1). IMD is associated with a case fatality rate (CFR) of 4.1%–20.0% (2) and long-term sequelae in up to 25% of survivors, such as neurological or hearing impairment, chronic pain, scarring, and amputation (3–5). Estimations of the incidence of IMD in the Asia-Pacific region are limited by inadequate population-based surveillance systems in most countries (6). In Taiwan, IMD is a notifiable disease, and hospitals are obliged to report cases to the Taiwan Centers for Disease Control (CDC). Between 1993 and 2020, 380 IMD cases were reported (7), but PCR positivity is not included in the diagnostic criteria used in Taiwan (8), so the true number of IMD cases is likely higher (9, 10).

Globally, 12 Nm serogroups have been identified, with six (NmA, NmB, NmC, NmW, NmX, and NmY) responsible for most IMD cases (11). In Taiwan, Nm serogroup B (NmB) disease was predominant in 1993–2020; overall, NmB was identified in 66% of recovered isolates and in 81% of isolates collected in 2003–2020 (7). This increase in NmB isolates was accompanied most notably by a decrease in NmW isolates (35% of recovered isolates in 1996–2002 versus 2% in 2003–2020). NmB IMD is prevalent in various Asia-Pacific countries (12). In China, results of a meta-analysis suggested that the proportion of NmB disease cases was 30% in 2010–2020 and an increase in incidence during this period, with 52% of cases in 2015–2020 caused by NmB (13). In Japan, between 2003 and 2020, NmB caused 26% of 188 IMD cases (14), while in South Korea, NmB accounted for 37% of 19 IMD cases in 2010–2016 (15). In Vietnam, a study of military hospitals between 2014 and 2021 found that 91% of 69 IMD cases were caused by NmB (16).

Two protein-based meningococcal serogroup B (MenB) vaccines have been licensed: the 4CMenB vaccine (Bexsero, GSK) (17, 18) and MenB-FHbp vaccine (Trumenba, Pfizer), which contains two of the three factor H-binding protein (fHbp) variants (subvariants 3.45 and 1.55) (19). 4CMenB includes three recombinant protein antigens, *Neisseria* adhesin A (NadA, peptide 3.8), neisserial heparin-binding antigen (NHBA, peptide 2), and fHbp (subvariant 1.1), plus detergent-extracted outer membrane vesicle (OMV) obtained from a New Zealand outbreak isolate, containing porin A protein (PorA, serosubtype 1.4) as the main vaccine antigen (17, 20). Bexsero is the only MenB vaccine available in Taiwan, where it was licensed for the immunization of individuals aged 2 months and older in May 2021 (21).

The licensure of MenB vaccines relied on safety data and immunogenicity results generated by the serum bactericidal antibody assay using human complement (hSBA assay), testing against vaccine antigen-specific indicator strains (22). As well as demonstrated immunogenicity, estimation of vaccine strain coverage enables understanding of the potential performance of MenB vaccines. Strain coverage by 4CMenB has been predicted using the Meningococcal Antigen Typing System (MATS) and genetic MATS (gMATS) (23–25). The latter can be performed using genome sequencing data for Nm from cultivable and non-cultivable clinical isolates, while MATS cannot be used for non-culture confirmed IMD cases. MATS data from more than 3,000 isolates from 17 countries were used to calibrate gMATS (25).

In the serogroup analysis of Nm isolates collected in Taiwan in 2003–2020, 134 NmB isolates were identified (7). Here, the NmB isolates are characterized by whole-genome sequencing (WGS) and vaccine antigen genotyping, and 4CMenB vaccine strain coverage is predicted using gMATS.

## RESULTS

### Distribution of NmB isolates

**Fig 1:**
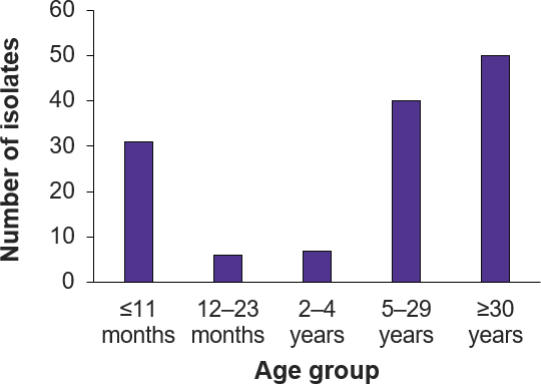
Distribution of 134 NmB isolates by patient age group.

Of the 134 NmB isolates, 83 (61.9%) were collected in the period 2003–2008, 20 (14.9%) in 2009–2014, and 31 (23.1%) in 2015–2020. Forty-four NmB isolates (32.8%) were from children aged under 5 years, of which 31 (23.1%) were from children aged under 12 months, while 40 (29.8%) were from individuals aged 5–29 years, and 50 (37.3%) isolates were from individuals aged 30 years or older (Fig. 1).

### Genetic characteristics of NmB isolates

**Fig 2:**
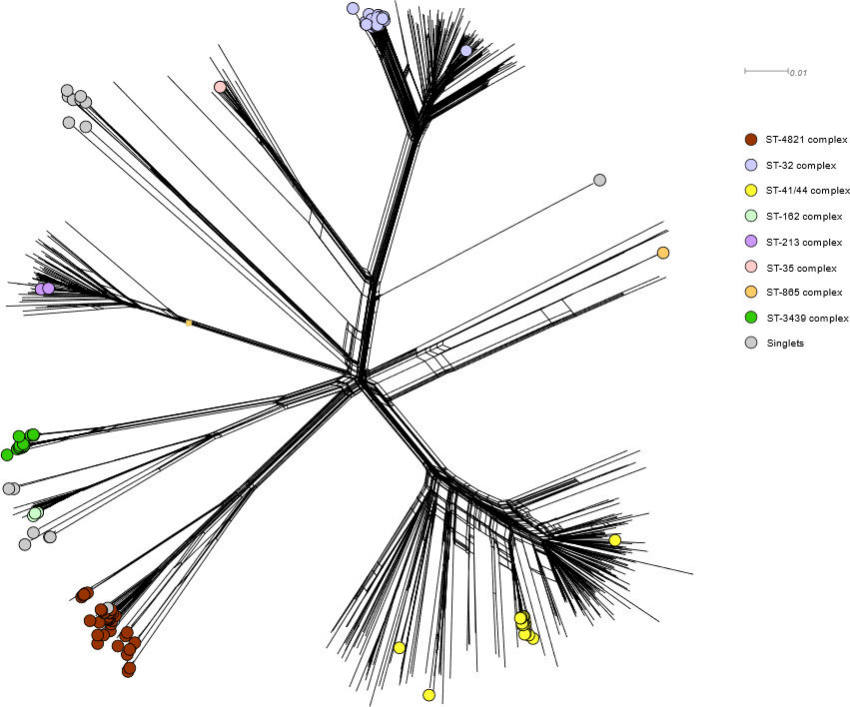
Phylogenetic distribution of clonal complexes of 134 NmB isolates from Taiwan in relation to 502 randomly selected NmB isolates genomes downloaded from PubMLST, as reconstructed in network created in SplitsTree using the NeighborNet algorithm based on single-nucleotide polymorphisms identified by kSNP algorithm.

The phylogenetic distribution of clonal complexes of the 134 NmB isolates, relative to 502 randomly selected NmB isolate genomes downloaded from PubMLST, was reconstructed in a network created in SplitsTree using the NeighborNet algorithm. Excluding singlets (i.e., profiles not assigned to a clonal complex: 18 isolates), eight clonal complexes were represented (Fig. 2). The most prevalent was sequence type (ST) 4821 complex (37 isolates, 27.6%), followed by ST-32 complex (32 isolates, 23.9%), ST-41/44 complex (20 isolates, 14.9%), and ST-3439 complex (17 isolates, 12.7%).

### Vaccine antigen genotyping

Reconstructions created from phylogenetic network analysis of 499 fHbp peptides, 508 NHBA peptides, and 130 NadA peptides downloaded from PubMLST and corresponding to NmB genomes only were superimposed with the peptides present in the 134 NmB isolates (Fig. S1 to S3). The prevalence of fHbp variants 1, 2, and 3 among the NmB isolates was 28.4% (38 isolates), 63.4% (85 isolates), and 4.5% (6 isolates), respectively (data not available for five isolates) (Fig. S1). The most common fHbp peptides were peptides 1.13, 2.16, 2.19, and 2.101. The NHBA peptides in the 134 NmB isolates were widely distributed among the global range of NHBA peptides (Fig. S2). The most common NHBA peptides were peptides 528 (31 isolates; 23.1%), 669 (27; 20.1%), 2 (17; 12.7%), and 803 (12; 9.0%).

Thirty-two Taiwan NmB isolates (23.9%) contained NadA peptides; Fig. S3 shows the results of the phylogenetic network analysis of NadA peptides in the isolates. For PorA, 11 (8.2%) isolates harbored PorA VR2 matching with peptide 4, with the remaining 123 isolates containing a diversity of other PorA peptides (see next section).

### Predicted MenB strain coverage

**Fig 3:**
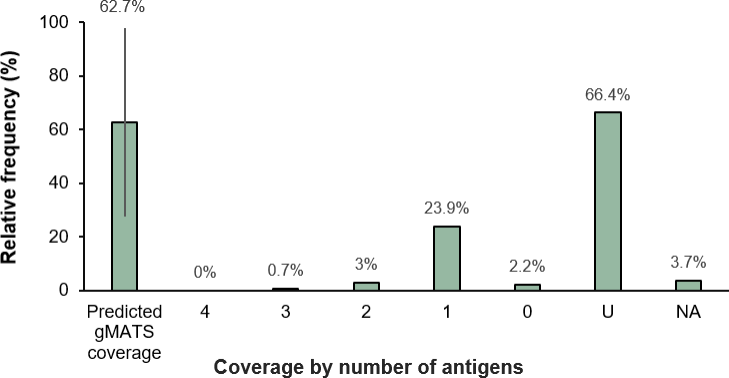
gMATS-based coverage distribution of isolates by the number of 4CMenB vaccine antigens contained in isolate contributing to coverage. Overall, 27.6% of isolates were covered, 2.2% were not covered, and 66.4% were unpredictable by gMATS. 4CMenB, 4-component MenB vaccine; U, unpredictable by gMATS; NA, genotyping data not available.

The predicted 4CMenB vaccine strain coverage by gMATS for the 134 isolates was 62.7% [lower limit (LL), 27.6%; upper limit (UL), 97.8%] (Fig. 3). Overall, 27.6% of isolates were covered, 2.2% were not covered, and 66.4% were unpredictable by gMATS (data not available for 3.7% of isolates) (Fig. 3). Only 29.8% of isolates were therefore predictable (covered/not covered) by gMATS. Coverage by number of antigens showed that the proportion of isolates predicted to be covered by gMATS was highest (23.9%) for isolates with one 4CMenB vaccine antigen (Fig. 3).

The gMATS coverage point estimates for each time period were 66.3% (LL, 33.7%; UL, 98.8%) for 2003–2008, 65.0% (LL 30.0%; UL 100%) for 2009–2014, and 51.6% (LL 9.7%; UL 93.5%) for 2015–2020. Analysis by Pearson’s chi-squared test showed no statistically significant differences in coverage point estimate by time period (data not shown). For each age group, gMATS coverage point estimates were 66.1% (LL 35.5%; UL 96.8%) for children younger than 12 months; 50.0% (LL 0%; UL 100%) and 42.9% (LL 14.3%; UL 71.4%) for children aged 12–23 months and 2–4 years, respectively; and 63.8% (LL 27.5%; UL 100%) and 64.0% (LL 28.0%; UL 100%) for individuals aged 5–29 years and 30 years or older, respectively.

**Fig 4:**
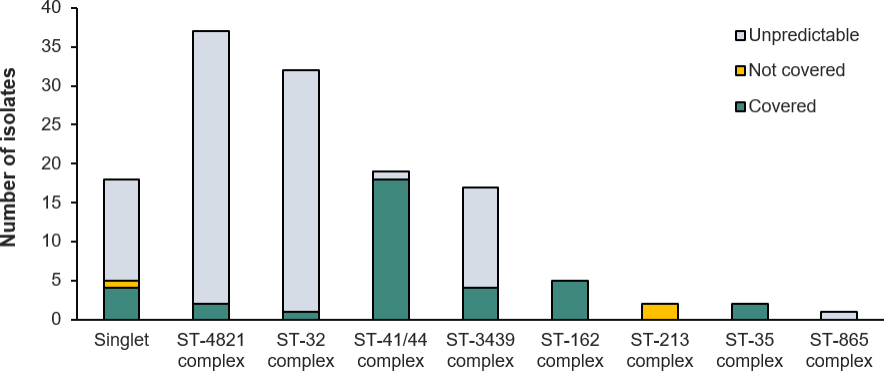
gMATS-based coverage distribution of isolates by clonal complex. ST, sequence type.

The analysis of gMATS coverage by clonal complex showed isolates with ST-41/44 complex contributed most to gMATS coverage (18 out of 20 isolates were covered), with contributions also from ST-162 (five of five isolates), ST-3439 (four of 17 isolates), ST-35 (two of two isolates), ST-4821 (two of 37 isolates), and ST-32 (one of 32 isolates) complexes (Fig. 4). gMATS point estimates for the complexes were 97.5% (LL, 95.0%; UL, 100%) for ST-41/44, 100% for ST-162 and ST-35, 61.8% (LL 23.5%; UL 100%) for ST-3439, 52.7% (LL 5.4%; UL 100%) for ST-4821, and 51.6% (LL 3.1%; UL 100%) for ST-32. However, gMATS coverage predictions for several clonal complexes were affected by high proportions of unpredictable isolates (76.5% unpredictable for ST-3439; >90% unpredictable for ST-4821 and ST-32).

**Fig 5:**
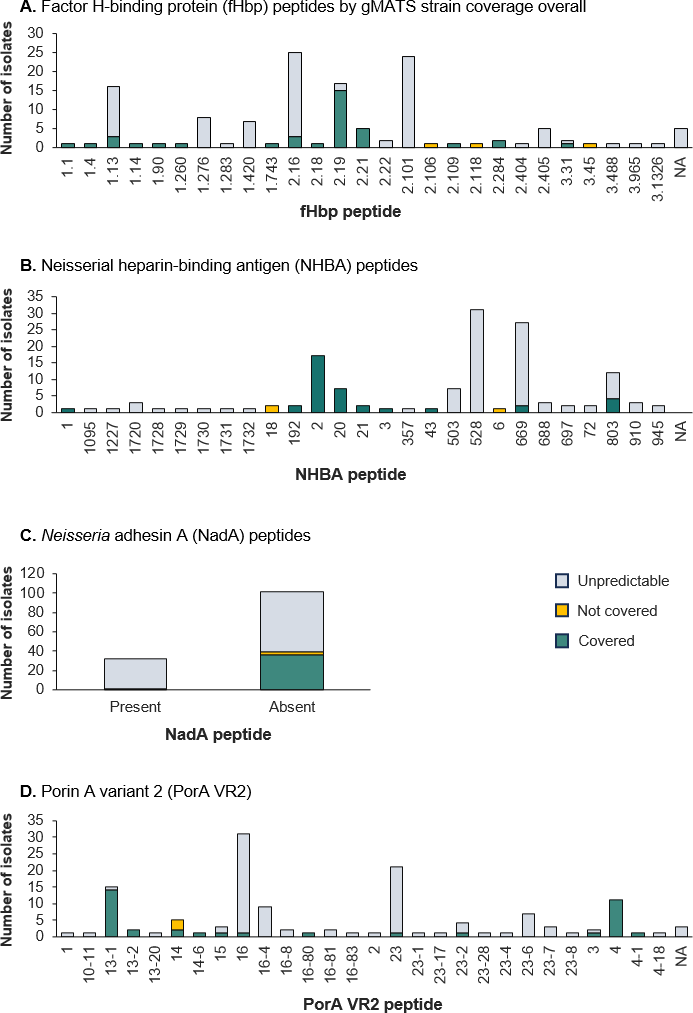
gMATS-based coverage distribution of isolates for individual 4CMenB vaccine antigen variants or peptides by gMATS strain coverage overall. 4CMenB, 4-component MenB vaccine; NA, genotyping data not available.

The analysis of single 4CMenB antigen coverage by gMATS showed coverage for four fHbp variant 1 peptides (peptides 1.1, 1.4, 1.14, and 1.90) (Fig. S4A). Analysis of data according to coverage of isolates containing at least one gMATS-positive antigen showed predicted coverage for isolates containing one of 14 fHbp peptides, including peptides belonging to fHbp variants 2 and 3 (Fig. 5A).

Results for the NHBA antigen indicated coverage for five peptides (peptides 1, 2, 20, 21, and 3) and a high rate of unpredictable coverage for other antigen peptides (Fig. S4B) but, for isolates with at least one gMATS-positive antigen, coverage was detected for nine NHBA peptides (Fig. 5B). For NadA, gMATS does not consider this 4CMenB antigen as there is no robust correlate with the NadA MATS outcome (as explained in Materials and Methods). Taking into account isolates containing at least one gMATS-positive antigen, among the 32 isolates with NadA peptides, one was covered, and among 102 isolates without NadA peptides, 36 had predicted coverage by gMATS, three were not covered, and the remainder had unpredictable coverage (Fig. 5C).

Coverage was demonstrated for 11 isolates harboring PorA VR2 matching with peptide 4, the exact match for the 4CMenB vaccine antigen. Analysis according to coverage by at least one gMATS-positive antigen predicted coverage for an additional 26 isolates with 11 other PorA VR2 peptides (Fig. 5D).

## DISCUSSION

This is the first study to evaluate the predicted coverage of NmB strains by 4CMenB in Taiwan, which was shown to be 62.7% using the genome-based tool, gMATS. Almost two-thirds of isolates were recovered in 2003–2008, followed by a drop in number in the second 6-year period. A subsequent increase in 2015–2020 reflected a reported increase in NmB disease in China during the same period (13). Approximately one-third of isolates were from children aged under 5 years, most of which were from infants (under 1 year old), a critical age-based risk group for MenB vaccination (26).

It was reported previously that NmB was the predominant serogroup in Taiwan in 2003–2020, and its prevalence had increased from 50.0% of isolates in 1996–2002 to 81.2% in 2003–2020 (7). In our analysis, 86.6% of NmB isolates from Taiwan were represented by eight clonal complexes, most commonly one of three global hyperinvasive clonal complexes, ST-4821, ST-32, and ST-41/44 (7, 27), and a newly assigned clonal complex, ST-3439 (7). ST-4821 was the leading NmB clonal complex, and this is also a major clonal complex group in China (7, 27, 28), where ST-4821 was initially identified in NmC strains, followed by NmB strains through capsular switching (29–31). An analysis of 378 NmB strains isolated in China between 2005 and 2016 found that ST-4821 was the most prevalent lineage in both patient-derived (37.9%) and healthy carrier-derived (35.6%) strains (32). In Taiwan, most ST-4821 isolates (40 of 50 ST-4821 Nm isolates) were NmB. ST-4821 and ST-32 were identified previously in association with two IMD outbreaks in Taiwan, one in a junior high school, and another at a military base (7). Outside of Asia, ST-32 and ST-41/44 were found to be the most common clonal complexes in an analysis of invasive NmB isolate panels from the United States, Australia, Canada, and nine European countries (33). In Taiwan, the percentages of NmB isolates assigned to ST-32 and ST-41/44 were higher than reported in China (2005–2016; 1.7% and 3.4%, respectively) (32).

The prevalences of fHbp variants 1 and 2 in our study are similar to those (32.1% and 61.9%, respectively) reported in a study of fHbp variants in 84 NmB patient strains isolated in China up to 2016 (34). Interestingly, in the same study, an examination of 445 NmB isolates derived from healthy carriers showed a 90.7% prevalence of fHbp variant 2 (34). Another study in China reported a higher proportion of variant 1 in NmB isolates from patients than from healthy carriers, suggesting that fHbp variant 1 correlates positively with NmB pathogenicity (32). Studies conducted in other regions show differences in the distribution of fHbp variants, with variant 1 predominant in invasive NmB isolate panels from the United States, Australia, Canada, and Europe (33). This highlights the importance of continuous monitoring to identify possible changes in the circulation of fHbp variants and new subvariants.

The NHBA peptides identified in the NmB isolates from Taiwan reflected the global distribution of peptides. Of the six most represented NHBA peptides in Taiwan, four (2, 20, 503, and 669) were also identified in the study in China (32). Two of the gMATS unpredictable peptides (528 and 669) are in the same cluster as NHBA peptide 2 (so matched to the vaccine). Almost one-quarter of isolates were positive for NadA peptide, which was similar to the percentage (27%) reported in a study of a global NmB strain panel (25) but higher than reported in NmB isolates from China, where 8% of 432 isolates were PCR-positive for the *nadA* gene (32). In Taiwan, we found that few isolates harbored the PorA variant contained in the 4CMenB vaccine, with high diversity in PorA, as reported in NmB isolates from China (32).

The predicted 4CMenB strain coverage by gMATS has a degree of uncertainty (LL 27.6%; UL 97.8%) that reflects the large proportion of unpredictable isolates by gMATS. However, half of gMATS unpredictable isolates are considered as covered by gMATS (25), and, in our study, the proportion of isolates predicted as not covered by 4CMenB was low (2.2%). The 62.7% gMATS point estimate is in line with gMATS estimates of between 58% and 91% reported in studies conducted in Europe, North America, and Australia (25, 35–41). Moreover, the 4CMenB coverage of NmB strains recovered in 2005–2016 in China was 63.6%, in terms of isolates containing one or two matching 4CMenB variants (32). In our study, among the different age groups, 4CMenB strain coverage by gMATS was highest for infants.

Interpretation of the predicted strain coverage needs to take into account that, overall, 66% of isolates were categorized as unpredictable, with predictable gMATS coverage for only 29.8% of NmB isolates. However, the gMATS-unpredictable percentages in the present analysis were highly variable, particularly in isolates positive for one antigen (fHbp, NHBA, or PorA) versus isolates gMATS-positive for one or more antigens. This suggests that unpredictable peptides may contribute to protection individually or in association with other 4CMenB antigens. Indeed, there is evidence that 4CMenB elicits antibodies against multiple surface-exposed antigens, which may act in concert and be functional against meningococcal strains not predicted to be covered (42). Different OMV components may also assist in providing protection since, while PorA is the immunodominant antigen (17, 20), OMV contains a complex mixture of antigens (43). Antibodies induced by fHbp, NHBA, and minor OMV components can bind the bacterial surface simultaneously, overcoming limitations of low surface expression and high antigenic diversity and triggering complement-mediated bacterial lysis (42). gMATS also underestimates the contribution of NadA antigen to coverage (44) and has specific limitations connected with the genotype–phenotype association approach (25). Underestimation of coverage with gMATS was demonstrated in an analysis of 40 isolates representative of IMD in England and Wales (25). Along with all gMATS-covered strains, 57% of gMATS-negative strains and 75% of gMATS-unpredictable strains were killed by hSBA. This analysis also showed a lower estimate of strain coverage by gMATS (72%–73%) than by hSBA assay (88%) (25, 45), which is a more precise measurement of coverage but not feasible for analyzing large numbers of NmB strains (46). Ultimately, the true impact and effectiveness of MenB vaccines can only be confirmed through real-world evidence of clinical outcomes. For 4CMenB, studies of real-world effectiveness in various age groups and with different vaccination schedules report effectiveness estimates of up to 94% against NmB disease (47).

In conclusion, the results of this analysis of NmB isolates from Taiwan show overall 4CMenB strain coverage of 62.7% by gMATS, which is in line with estimates from other countries, but coverage was predictable for only 29.8% of isolates. These are likely to be underestimates since, similar to MATS, gMATS does not measure the contribution to killing of synergistic mechanisms associated with simultaneous binding of antibodies elicited by multicomponent vaccines, such as 4CMenB, to multiple antigenic targets. The contribution of NadA and minor OMV vaccine components is also not taken into consideration with gMATS. Nevertheless, these results contribute to better understanding of disease-causing NmB strains in Taiwan and indicate the need to monitor their epidemiology.

## MATERIALS AND METHODS

### Nm isolates

As explained previously (7), 165 Nm isolates were recovered between 2003 and 2020 from the blood of patients with IMD and obtained from the Biobank Section of the Taiwan CDC. A total of 134 NmB isolates underwent genome sequencing and assembly from the raw sequencing reads provided by author Dr. Min-Chi Lu (7). Genome assembly was conducted with the Unicycler v.0.4.9b pipeline based on SPAdes assembler (48).

### Phylogenetic analysis

WGS of bacterial isolates was conducted by the Taiwan CDC, and 4CMenB antigen typing was used to generate allelic profiles for the 134 NmB isolates, as described previously (7).

We characterized the whole-genome diversity of the Taiwan NmB isolates in terms of their phylogenetic relationships with a sampled subset of 502 NmB isolate genomes downloaded in September 2022 from the PubMLST Neisseria database (https://pubmlst.org/organisms/neisseria-spp). The 502 NmB isolates were randomly selected from the PubMLST list of >8,000 NmB genomes, representing the global collection of genomes archived in the database.

Phylogenetic reconstructions were made using NeighborNet method (49) computed by SplitsTree software (version 4.14) (50) and based on the sequences alignment of single-nucleotide polymorphisms computed by kSNP (version 4.0) algorithm.

### Vaccine strain coverage prediction by genotyping

The *fHbp*, *nhba*, *nadA*, and *porA* genes and their protein translations were extracted from the whole-genome sequences by BIGSdb application (version 1.24) using default settings (51). Alleles and corresponding peptide identification numbers (IDs; protein variants) were assigned using the PubMLST *Neisseria* species database definitions. Antigen-specific strain coverage predicted by gMATS was defined by identifying peptide IDs significantly associated with MATS coverage/non-coverage for that antigen, as described previously (25) and shown in Table S1.

For fHbp and NHBA antigens, peptide IDs present in more than five isolates were considered. Peptide IDs for which the percentage of MATS-covered strains was higher than 60% or lower than 40% were considered predictors of coverage or non-coverage, respectively, if a test of proportions rejected 50% as null hypothesis (*P* < 0.05 or <0.001). Peptide IDs not fulfilling these criteria were considered unpredictable. The same approach was attempted for NadA, testing the association of *nadA* gene presence/absence, and NadA-MATS coverage. However, the information for this antigen, limited to its presence/absence, failed to establish a robust correlate with NadA MATS outcome. For this reason, the contribution of NadA antigen to the gMATS coverage estimation was disregarded. The contribution to coverage by the OMV component was estimated by sequencing part of the *porA* gene encoding variable region 2 (VR2) and checking identity to the variant present in the vaccine, i.e., PorA VR2 match with peptide 4 was defined as covered (PorA VR2 = 4) and other cases as not covered (23).

An isolate was defined as gMATS-covered if one or more antigen-specific predictions for that strain were covered. If all antigen-specific gMATS predictors were not covered, the isolate was defined as gMATS not covered. All remaining isolates were defined as gMATS unpredictable. Previous gMATS coverage data on over 3,000 MenB isolates found that 49% of gMATS unpredictable isolates were MATS-covered (25), so half of gMATS unpredictable isolates were considered as covered by gMATS in this analysis.

## Data Availability

Data used for this publication were generated by CDC. For access to anonymized subject-level data, please contact CDC or Min-Chi Lu (luminchi@gmail.com).

## SUPPLEMENTAL MATERIAL

Supplemental material msphere.00220-24-s0001.docx Supplemental table and figures.

ASM does not own the copyrights to Supplemental Material that may be linked to, or accessed through, an article. The authors have granted ASM a non-exclusive, world-wide license to publish the Supplemental Material files. Please contact the corresponding author directly for reuse.
