# Supplementary material for: Prediction by genetic MATS of 4CMenB vaccine strain coverage of invasive meningococcal serogroup B isolates circulating in Taiwan between 2003 and 2020
Source: mSphere. 2024 May 16;9(6):e00220-24. doi: 10.1128/msphere.00220-24 (PMC11338074; doi:10.1128/msphere.00220-24)
Supplement: Supplemental material — Supplemental table and figures. [file msphere.00220-24-s0001.docx]

# SUPPLEMENTARY MATERIAL

**Table S1.** The genetic Meningococcal Antigen Typing System (gMATS) predictors identified via analysis of associations between antigen genotype and MATS coverage (1).

| **4CMenB antigen** | **gMATS predictors** | | |
| --- | --- | --- | --- |
|  | **Covered** | **Not covered** | **Unpredictable** |
| fHbp | Peptides 1, 2, 4, 14, 15, 37, 89, 90, 110, 144, 224, 232, 245, 249, 252, 510 | Peptide 213 and all variant 2 and 3 peptides | All other fHbp variant 1 peptides |
| NHBA | Peptides 1, 2, 3, 5, 10, 20, 21, 113, 243 | Peptides 6, 13, 17, 18, 19, 24, 25, 30, 31, 43, 47, 58, 112, 114, 120, 122, 160, 187, 253 | All other NHBA peptides |
| NadA | Never | Always | Not applicable |
| OMV | PorA VR2 = 4 | PorA VR2 ≠ 4 | Not applicable |

4CMenB, 4-component meningococcal serogroup B vaccine; fHbp, factor H binding protein; NHBA, neisserial heparin-binding antigen; NadA, *Neisseria* adhesin A; OMV, outer membrane vesicles; PorA VR2, porin A variable region 2.

Peptide numbers correspond to identification numbers in PubMLST *Neisseria* sequence typing database.

**Reference**

1. Muzzi A, Brozzi A, Serino L, Bodini M, Abad R, Caugant D, Comanducci M, Lemos AP, Gorla MC, Křížová P, Mikula C, Mulhall R, Nissen M, Nohynek H, Simões MJ, Skoczyńska A, Stefanelli P, Taha MK, Toropainen M, Tzanakaki G, Vadivelu-Pechai K, Watson P, Vazquez JA, Rajam G, Rappuoli R, Borrow R, Medini D. 2019. Genetic Meningococcal Antigen Typing System (gMATS): A genotyping tool that predicts 4CMenB strain coverage worldwide. Vaccine 37:991-1000.

**FIG S1** Phylogenetic network analysis of 499 factor H-binding protein (fHbp) peptides downloaded from PubMLST, showing (with blue dots) the peptides present in the 134 NmB isolates from Taiwan


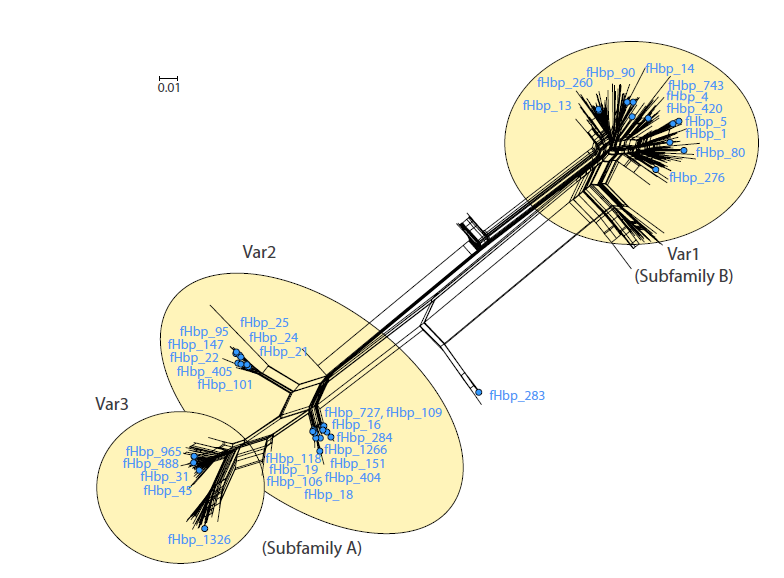


NmB, *Neisseria meningitidis* serogroup B; Var, variant

**FIG S2** Phylogenetic network analysis of 508 neisserial heparin-binding antigen (NHBA) peptides downloaded from PubMLST, showing (with blue dots) the peptides present in the 134 NmB isolates from Taiwan


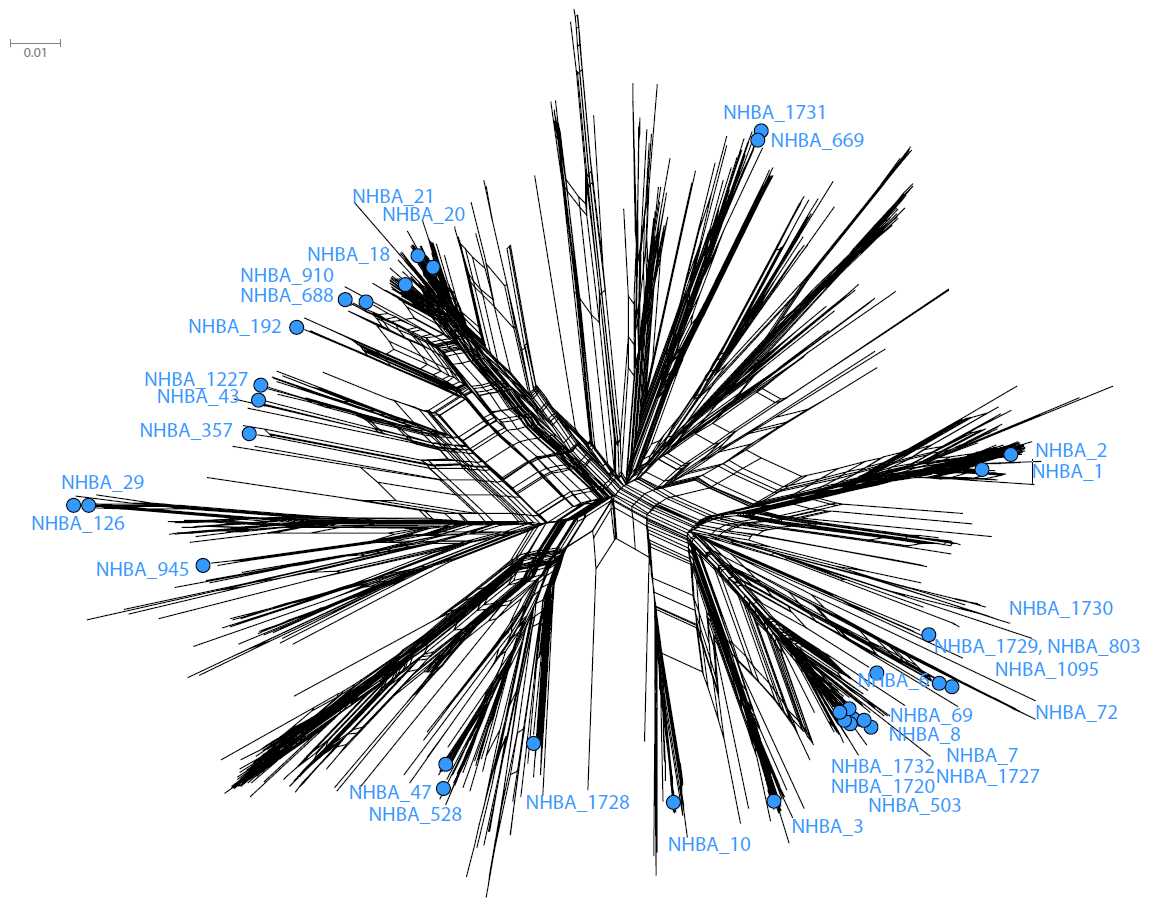


NmB, *Neisseria meningitidis* serogroup B

**FIG S3** Phylogenetic network analysis of 130 *Neisseria* adhesin A (NadA) peptides downloaded from PubMLST, showing (with blue dot) presence in the 134 NmB isolates from Taiwan


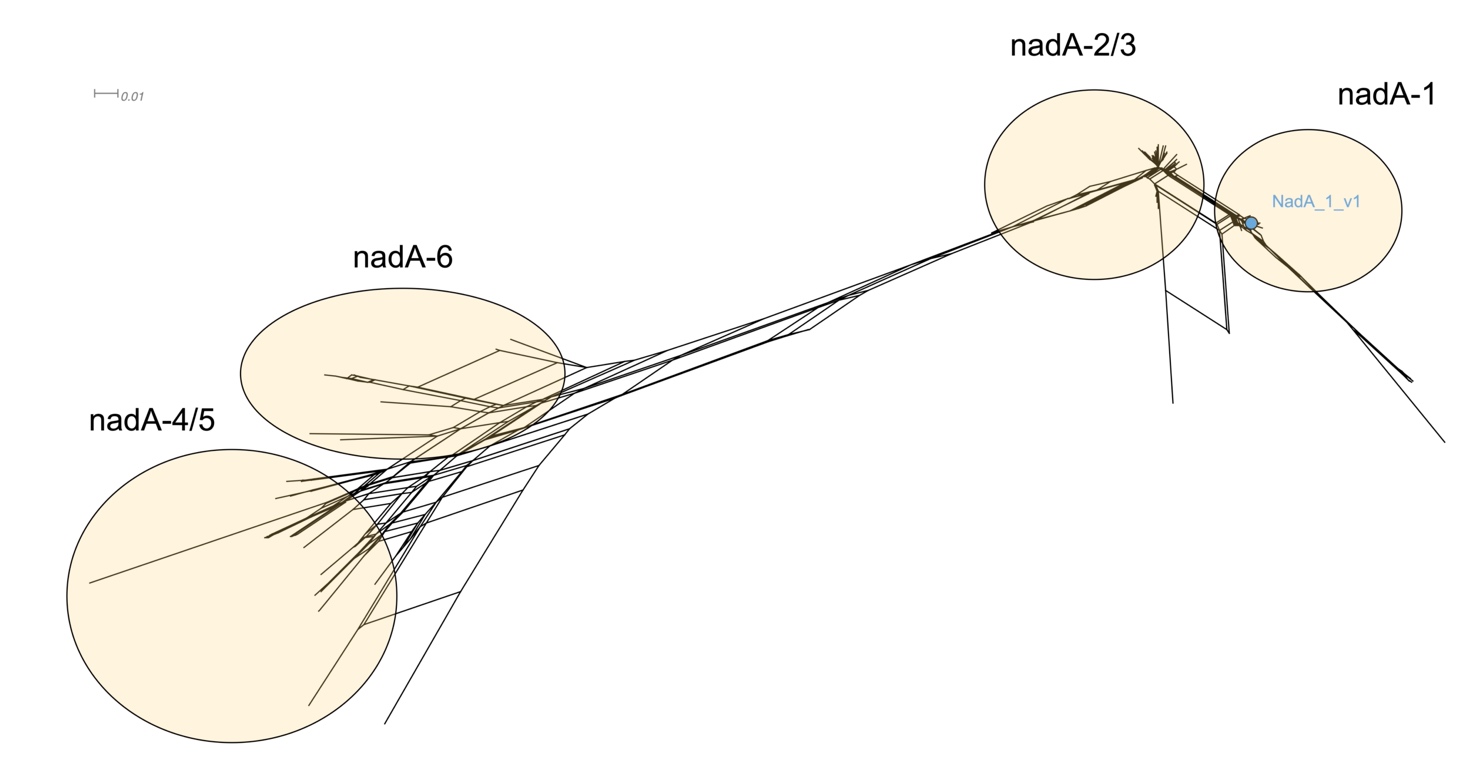


NmB, *Neisseria meningitidis* serogroup B

**FIG S4** Genetic Meningococcal Antigen Typing System (gMATS)-based coverage distribution of isolates by individual 4CMenB vaccine antigens (peptides).

A. Factor H-binding protein (fHbp) peptides by fHbp gMATS coverage


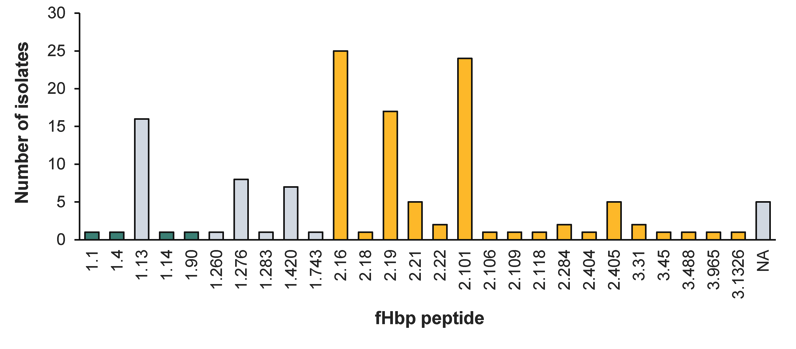


B. Neisserial heparin-binding antigen (NHBA) peptides by NHBA gMATS coverage


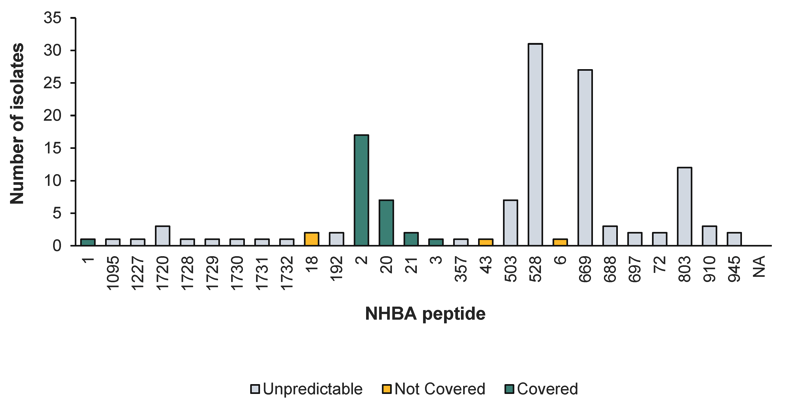


4CMenB, 4-component meningococcal serogroup B vaccine; NA, genotyping data not available
